# Real-time polymerase chain reaction optimised for hepatitis C virus detection in dried blood spots from HIV-exposed infants, KwaZulu-Natal, South Africa

**DOI:** 10.4102/ajlm.v5i1.269

**Published:** 2016-03-18

**Authors:** Anneta Naidoo, Raveen Parboosing, Pravi Moodley

**Affiliations:** 1 Department of Virology, Nelson R Mandela School of Medicine, School of Laboratory Medicine and Medical Sciences, University of KwaZulu-Natal, Durban, KwaZulu-Natal, South Africa; 2 National Health Laboratory Services, Inkosi Albert Luthuli Central Hospital, Durban, KwaZulu-Natal, South Africa

## Abstract

**Background:**

There is a paucity of data on the prevalence of hepatitis C virus (HCV) in children, particularly in sub-Saharan Africa. A major obstacle in resource-limited settings for polymerase chain reaction (PCR) testing is the necessity for specimen transportation and storage at low temperatures. There are numerous recent studies of using real-time HCV PCR for diagnosis and screening of plasma and serum, but few have looked at using dried blood spot (DBS) specimens.

**Objectives:**

The aim of this study was to optimise a real-time HCV PCR method to detect HCV RNA from infant DBS specimens for use as a tool for HCV surveillance in KwaZulu-Natal, South Africa.

**Method:**

The LightCycler^®^ 2.0 instrument was used for the HCV PCR using the LightCycler^®^ RNA Master SYBR Green I kit. Template volume, primer concentration and primer annealing temperatures were optimised and the method was used on 179 DBS specimens from HIV-exposed infants in KwaZulu-Natal.

**Results:**

Primer concentrations adjusted to 0.25 µM and a template volume of 10 µL improved the PCR amplification. Primer annealing temperatures lowered from 65 °C to 58 °C resulted in higher quantities of amplified PCR product. The limit of detection of the optimised HCV PCR assay was between 1200 IU/mL and 3580 IU/mL of HCV RNA. HCV was not detected in any of the 179 DBS specimens.

**Conclusion:**

The optimised real-time HCV PCR on infant DBS specimens performed well, but HCV was not found in this surveillance study. HIV infection may have little impact on the vertical transmission of HCV in this region.

## Introduction

Hepatitis C virus (HCV) infects 3% of the global population,^1^ but there is a paucity of data on HCV prevalence in children, particularly children in sub-Saharan Africa.^2^ HCV is usually transmitted to infants vertically, with a rate that varies from 3% to 7%, although it may increase two- to five-fold with HIV co-infection.^3^ The prevalence of HCV is higher amongst HIV-positive patients because of shared routes of transmission and common risk factors.^4^ The estimated prevalence of HCV in sub-Saharan Africa was 3% amongst HIV-negative individuals in 2002, but prevalence increased to 7% amongst individuals with HIV co-infection by 2014.^5^ The prevalence of HCV/HIV co-infection in South Africa was 0.1% in 2002^6^ compared to 3.5% amongst individuals infected only with HCV.^7^ There are reasons to suspect that the prevalence of HCV may be high in KwaZulu-Natal, South Africa. Firstly, KwaZulu-Natal has a high antenatal HIV prevalence of 40.1%.^8^ Secondly, KwaZulu-Natal has a high overall adult HCV prevalence of 6.4% and a much higher prevalence of HCV/HIV co-infection (13.4%),^9^ which may imply a high prevalence of HCV amongst pregnant woman. The World Health Organization suggests that screening for HCV in regions with a high prevalence of HIV should be conducted, but depends on HCV prevalence and resources.^10^

In a region with a high prevalence of both HIV and HCV, especially amongst pregnant women,^9^ it is important to determine the prevalence of HCV amongst infants. Although most children with chronic HCV infection remain asymptomatic, some may have mild abnormalities in liver function and chronic HCV infection may progress to severe liver disease, end-stage liver disease and death.^2^ Liver disease resulting from HCV infection is a major cause of mortality and morbidity amongst adults co-infected with HIV and HCV, which further compounds the hepatotoxic effects of antiretroviral therapy.^4^

Infant diagnosis of HCV by molecular-based technologies is superior to serological techniques for detecting HCV infection by means of maternal antibodies circulating in infants.^2^ A major obstacle in resource-limited settings for polymerase chain reaction (PCR) testing is the necessity of transporting and storing specimens at low temperatures. However, using dried blood spot (DBS) specimens, which do not require refrigeration, significantly reduces pre-analytical problems and is widely used in molecular virology for the detection of viral nucleic acids.^11^

Numerous studies have reported on the use of real-time PCR for HCV diagnosis using plasma and serum,^12^^,^^13^^,^^14^^,^^15^^,^^16^^,^^17^^,^^18^^,^^19^^,^^20^ although few have looked at DBS specimens.^21^^,^^22^^,^^23^^,^^24^^,^^25^ However, a few recent studies have found good correlations for adult serum and plasma compared with DBS specimens analysed using commercial HCV PCR assays.^26^^,^^27^^,^^28^^,^^29^

The prevalence of HCV amongst HIV-exposed infants in KwaZulu-Natal, South Africa is unknown. Therefore, the aim of this study was to optimise a real-time PCR assay to detect HCV RNA from HIV-exposed infant DBS specimens for use as a tool for HCV surveillance in KwaZulu-Natal, South Africa.

## Research method and design

### Ethical considerations

Ethical clearance was obtained from the University of KwaZulu-Natal Biomedical Research Ethics Committee (BE273/090). Informed consent was not required since this was a retrospective study in which we used discarded specimens from routine testing. All data were recorded and then anonymised for the purposes of the study. Specifically, HIV PCR results, HCV PCR results, age and clinical diagnosis were recorded anonymously in a spreadsheet. No patient identifiers were recorded.

### Study design

This study was conducted retrospectively in the laboratory at the Department of Virology, National Health Laboratory Service/University of KwaZulu-Natal, at the Inkosi Albert Luthuli Central Hospital in Durban, South Africa. This is a reference laboratory which receives all DBS specimens from public sector facilities in KwaZulu-Natal for PCR testing of DNA from HIV-exposed infants for early diagnosis of HIV infection. The number of DBS specimens tested by the laboratory has steadily increased each year from 13 699 in 2005 to 73 033 in 2012. The uptake of HIV PCR testing amongst HIV-exposed infants in KwaZulu-Natal has also increased from 18% in 2005 to 97.4% in 2011.^30^ A sample size of 179 was calculated using Epi-Info™ 6 (Centre for Disease Control, Atlanta, Georgia, United States, 1998) using the number of live births in KwaZulu-Natal,^30^ the prevalence of HIV amongst antenatal attendees (which approximates the percentage of HIV-exposed infants)^8^ and the approximate HCV seropositivity in infants, based on data from the laboratory information system. The sample size was representative of HIV-exposed infants who attended public health facilities in KwaZulu-Natal and who required HIV PCR testing because of suspected HIV exposure or clinical suspicion of HIV infection.

### Specimen collection

As part of routine early infant diagnosis of HIV, infants’ blood is collected by heel-prick and spotted onto Whatman Filter paper in four circles. In the laboratory, two spots are cut out and used for routine HIV PCR testing within four to five days of DBS specimen collection. After releasing the results, the card (with the two unused spots) is routinely stored at room temperature for at least three months and then discarded. These discarded DBS specimens were tested for HCV after specimens had been stored for at least three months after initial collection. Specimens with inadequate volume (where the entire circle was not filled with blood) were excluded from the study. Acceptable DBS specimens were sequentially selected from August to December 2010 until 179 specimens were obtained. External quality control specimens were purchased from Quality Control for Molecular Diagnostics (QCMD; Glasgow, Scotland) and internal quality control specimens were prepared in-house.

### Laboratory methods

Before automated extraction, using the NucliSENS^®^ easyMag^®^ Extraction (biomérieux, Marcy I’Etoile, France), two DBS (approximately 50 µL blood per spot) were cut with sterilised scissors into a 1.5 mL eppendorf tube and 2 mL NucliSENS^®^ easyMAG^®^ lysis buffer containing 5 M guanidinium thiocyanate was added to the tube. The lysis buffer was also added to the quality control specimens. The specimens were agitated for 30 minutes on an orbital shaker and centrifuged at 1500 x g for two minutes. Supernatant was pipetted into transfer wells, which were then loaded onto the automated extraction system. Briefly, cell-bound nucleic acids were released with the chaotropic agent − guanidinium thiocyanate − and then bound to silica particles, which were immobilised on a filter. Impurities were removed by several washes in buffers containing guanidinium thiocyanate, 70% ethanol and acetone, after which the purified nucleic acids were eluted in molecular grade water.

The LightCycler^®^ 2.0 instrument (Roche Diagnostics, Mannheim, Germany) was used to perform reverse transcription and amplification in a one-step assay using the LightCycler^®^ RNA Master SYBR Green I kit (Roche Diagnostics, Mannheim, Germany), which contained the enzymes *Thermus thermophilus* DNA polymerase, dNTPs, reaction buffer and SYBR Green I fluorescent dye.

The primers used in the reverse transcription PCR were KY80 (sense) 5’-GCA GAA AGC GTC TAG CCA TGG CGT-3’ and KY78 (antisense) 5’-CTC GCA AGC ACC CTA TCA GGC AGT-3’. These primers target the highly-conserved 5’-non-coding region of the HCV genome to produce a PCR product of 244 bp and are known to detect all HCV genotypes.^31^ The reverse transcription PCR master mix consisted of PCR-grade water, Mn(OAc)_2_, both primers and the LightCycler^®^ RNA Master SYBR Green I mix.

The reverse transcription and PCR parameters were set according to the LightCycler^®^ RNA Master SYBR Green I package insert. Reverse transcription of the template RNA was performed at 61 °C for 20 minutes. Complementary DNA (cDNA) was amplified over 40 cycles of denaturation, annealing and extension. Denaturation and extension were performed at the temperatures on the package insert. The annealing temperature was calculated using the melting temperatures of the primers, namely, 65 °C (4 °C subtracted from 69 °C), using standard PCR optimisation guidelines, whereby a range of annealing temperatures (55 °C to 65 °C) was used to determine the optimal annealing temperature, where efficiency of PCR amplification was maximal.^32^ Melting curve analysis to identify PCR products was performed as described on the package insert. The PCR products were detected with a SYBR Green I dye, which intercalates with double-stranded DNA and is detected at a wavelength of 530 nm.

Primer concentrations and template volumes were optimised using the methods described on the LightCycler^®^ RNA Master SYBR Green I package insert. Primer concentrations ranging from 0.1 µM to 0.5 µM, using two-fold dilutions, were used to determine the optimal primer concentration capable of detecting the lowest concentration of HCV RNA. Template volumes of 1 µL, 5 µL and 10 µL were used to determine the optimal starting concentration of the template RNA.

The HCV Quantitative PCR proficiency panel (QCMD, Glasgow, Scotland) was prepared in five-fold dilutions as an external quality control standard to calculate the lower limit of detection of the optimised HCV RNA PCR assay.

For the preparation of the in-house internal quality control specimens, HCV-negative whole blood was provided by the South African National Blood Services (Durban, South Africa). These DBS specimens were prepared by spiking the HCV-negative whole blood with HCV-positive plasma (3 580 000 IU/mL) obtained from the National Institute of Communicable Diseases (Sandringham, Johannesburg, South Africa). This plasma was tested for HCV using the COBAS AmpliPrep/COBAS TaqMan Hepatitis C Virus assay on the COBAS AmpliPrep and COBAS TaqMan 48 analyser (Roche Diagnostics, Mannheim, Germany). Ten-fold serial dilutions were made, with a starting concentration of 3 580 000 IU/mL of HCV RNA, resulting in values of 3 580 000 IU/mL, 358 000 IU/mL, 35 800 IU/mL and 3580 IU/mL. Unspiked whole blood was used as a negative control. Fifty microliters (50 µL) of the positive and negative internal control specimens were spotted onto Whatman Filter paper to mimic infant DBS specimens.

## Results

**FIGURE 1:**
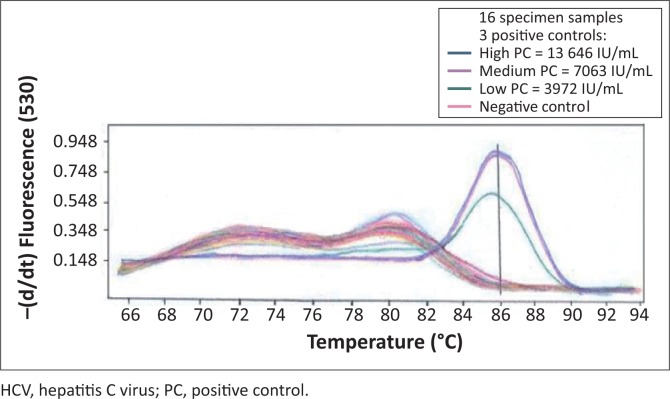
Melting curves at a melting temperature of 86 °C represent HCV-positive controls. Sixteen specimens that tested negative for HCV by serology were used as HCV-negative controls.

**FIGURE 2:**
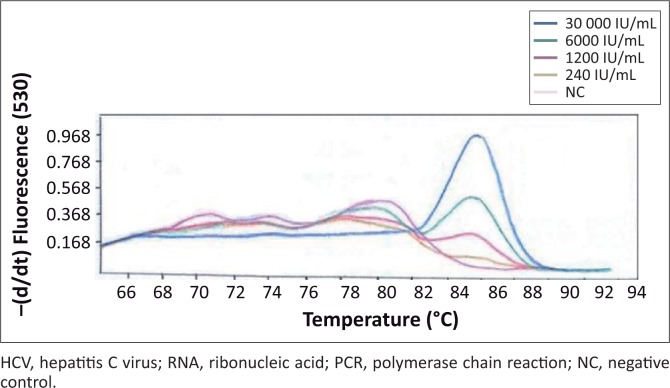
External quality controls. The melting curves show results of five-fold dilutions of HCV-positive external quality control specimens. These specimens were used to determine the lowest HCV RNA-positive concentrations that the optimised PCR assay could detect. HCV RNA at concentrations of 30 000 IU/mL, 6000 IU/mL and 1200 IU/mL were detected by the assay, whereas concentrations of 240 IU/mL of HCV RNA and lower were undetectable.

**FIGURE 3:**
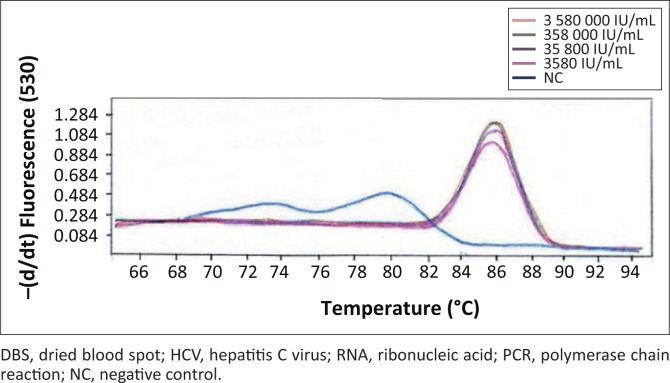
Internal quality controls. Internal quality control DBS specimens were prepared by spiking HCV-negative whole blood with HCV-positive plasma. Ten-fold dilutions were prepared with a starting concentration of 3580 000 IU/mL of HCV RNA and added onto Whatman Filter paper. HCV-negative whole blood was used as a negative control. An HCV RNA concentration of 3580 IU/mL was the lowest concentration of RNA detected for DBS using the optimised PCR assay.

Primer concentrations adjusted to 0.25 µM and a template volume of 10 µL improved PCR amplification. An annealing temperature of 58 °C resulted in higher levels of amplified PCR product compared with an annealing temperature of 65 °C. Quality control specimens with HCV RNA values of < 10 000 IU/mL were detectable at an annealing temperature of 58 °C. Detection at a melting temperature of 86 °C was indicative of an HCV-positive result (Figure 1) for quality control specimens with viral loads of 13 646 IU/mL (high), 7063 IU/mL (medium) and 3972 IU/mL (low). The external quality control specimens with known HCV viral loads of 30 000 IU/mL, 6000 IU/mL and 1200 IU/mL were detected by the PCR, whereas external quality control specimens with an HCV viral load of 240 IU/mL were undetectable (Figure 2). The in-house HCV quality control specimens with viral loads of 3 580 000 IU/mL, 358 000 IU/mL, 35 800 IU/mL and 3580 IU/mL were all detected by the HCV PCR (Figure 3).

The lowest concentration detected by the optimised HCV PCR was 1200 IU/mL (Figure 2) when using the QCMD quality control specimens. The in-house internal quality control DBS specimens were detected with viral loads as low as 3580 IU/mL of HCV RNA (Figure 3).

Of the 179 infant DBS specimens, 13 tested HIV-positive and 166 tested HIV-negative via PCR. All 179 DBS specimens tested negative for HCV using the optimised real-time PCR assay.

## Discussion

We optimised a real-time PCR test for HCV using DBS specimens from infants as a simple method for qualitative HCV detection. Although our method was qualitative, it was able to detect at least 3580 IU/mL and 1200 IU/mL in various types of quality control specimens. This could be useful for detecting acute HCV infections, which generally have viral loads less than 10^5^ IU/mL.^33^ The detection of amplified nucleic acid using SYBR Green I is inexpensive and is an easier technical approach in comparison to other real-time PCR formats such as TaqMan and molecular beacon detection methods.^14^ It is possible that HCV RNA viral load in our infant DBS specimens was below the level of detection of the assay, since HCV RNA viral loads amongst infants in the first year of infection may be too low for detection.^3^

DBS specimens have improved the cost-effectiveness, coverage and availability of molecular-based testing in resource-limited settings.^11^^,^^24^^,^^34^^,^^35^ This has resulted in the development of several optimised PCR techniques to detect HCV using DBS.^21^^,^^22^^,^^24^^,^^25^ As with our method, several of these techniques are based on real-time PCR and, like De Crignis,^22^ we used LightCycler^®^ technology with SYBR Green I as the fluorophore.^21^^,^^24^^,^^25^ Probe-based detection methods are known to improve the sensitivity of target detection, but increase the cost of the testing.^21^ The limit of detection varies amongst assays. Our assay had a limit of detection of 3580 IU/mL, which is higher than previously reported limits of detection of 2500 IU/mL^22^ and 250 IU/mL.^21^ This difference may be attributed to differences in storage conditions of the DBS specimens before testing and differences in methods of RNA extraction, which may result in variations in the number of copies of starting RNA.^11^^,^^25^^,^^34^ Additionally, DBS specimen volumes are lower when compared with plasma and serum specimens, which reduces the amount of RNA extracted.^34^ Thus, the correlation between HCV viral load from DBS and HCV viral load from plasma or serum may be imprecise, suggesting that DBS specimens are better suited to diagnosis, whereas plasma or serum are better suited to monitoring of HCV antiviral treatment.^34^ These methods for HCV testing using real-time PCR have been applied to HCV treatment monitoring,^24^ screening for HIV/HCV co-infection^22^ and surveillance for HCV amongst intravenous drug users.^36^ Our real-time HCV PCR was used on DBS specimens from an infant population, which may be useful for HCV surveillance amongst infants.

The use of DBS specimens for HCV PCR has several advantages.^11^^,^^34^^,^^35^ Firstly, they are easier to collect than whole blood and present a lower infectious hazard. Secondly, specimen transportation and storage are cheaper, since refrigeration is not required. Lastly, the long-term stability of nucleic acids in DBS has been proven for molecular analysis. Therefore, real-time HCV PCR using DBS specimens for HCV surveillance has been used to understand the molecular epidemiology of HCV.^21^^,^^22^^,^^23^^,^^24^^,^^25^^,^^36^

### Limitations

Our HCV PCR optimisation has several limitations. Firstly, the results are not representative of all infants, since only specimens from HIV-exposed infants who require an HIV PCR were tested. Secondly, our optimised HCV PCR may not be optimal for routine clinical diagnostic purposes, although it may be convenient for surveillance work. Thirdly, we did not compare our DBS specimen method to those using whole blood. However, we did use alternative methods to evaluate our PCR assay, which included testing external quality control specimens with known HCV viral loads ranging from 1081 IU/mL to 34 594 IU/mL and clinical HCV-positive and HCV-negative specimens from a reference laboratory. Further, positive and negative controls were included in each run. Lastly, we chose not to follow previously described methods but to optimise our own method, because it was more cost-effective than other real-time techniques reported. In addition, we used existing equipment and in-house PCR consumables that the technical staff in our laboratory were already proficient in using.

Given that our DBS specimens were from HIV-exposed infants in KwaZulu-Natal, we expected to find a moderate prevalence of HCV amongst these infants as reported by other African countries with populations co-infected with HIV and HCV.^37^^,^^38^^,^^39^ Infection with HIV is a risk factor for HCV transmission in co-infected pregnant women and is known to increase vertical transmission.^3^^,^^4^ The fact that we did not detect HCV RNA in our study specimens may be because of the prolonged storage (≥ 3 months) of our DBS specimens at room temperature, which may have resulted in degradation of HCV RNA.^25^

Furthermore, perinatal transmission of HCV requires a high maternal HCV viral load.^3^ Detection of HCV RNA at birth is usually indicative of intrauterine infection, whereas infection during delivery is associated with HCV RNA being detected only after infants are aged six months; in addition, delivery by caesarean section is less frequently associated with HCV transmission than vaginal delivery.^3^ However, clinical information, such as maternal HCV viral load, mode of delivery of infants, infant liver function markers, risk factors (such as HBV co-infection) and laboratory results (such as CD4 cell counts and HIV viral loads) were not available for our study.

## Conclusion

We have demonstrated that using an optimised real-time HCV PCR assay on DBS specimens from HIV-exposed infants is a good technique for HCV detection and may be well suited for surveillance purposes in resource-limited settings. To our knowledge, this is the first surveillance in South Africa using an optimised real-time HCV PCR to report the prevalence of HCV amongst HIV-exposed infants. Considering that HCV was not detected in our DBS specimens, HCV prevalence in this population of infants in KwaZulu-Natal, South Africa may be low and may have little impact on HIV-exposed infants. Therefore, further surveillance for HCV amongst HIV-exposed infants in our setting may not be necessary. However, HCV surveillance may still be prudent for HIV-exposed infants in countries with moderate to high HIV and HCV prevalence and should include collection of clinical information and data about confounding factors.
